# Rate and mechanism of thiolate deligation in Au_25_ nanoclusters *via in operando* electrochemical impedance spectroscopy

**DOI:** 10.1039/d5sc05597k

**Published:** 2025-11-12

**Authors:** Eric Z. Liu, Samuel D. Parker, Dylan P. Tietje-Mckinney, Miguel Orozco, Trevor W. Hayton, Lior Sepunaru

**Affiliations:** a Department of Chemistry and Biochemistry, University of California at Santa Barbara CA 93106 USA sepunaru@ucsb.edu

## Abstract

Nanomaterial electrocatalysis is a critical field for advancing sustainable energy technologies, yet determining the active catalytic species remains a significant challenge as the active species is often a result of dynamic structural evolution of the catalyst during the reaction. In this work, we investigate reductive deligation, a well-known activation process of ligated nanomaterials, on [Au_25_(PET)_18_]^−^ (PET = 2-phenylethanethiol) nanoclusters (Au_25_) under varying electrochemical conditions. We introduce a novel application of electrochemical impedance spectroscopy (EIS) to characterize the Au_25_*in situ* throughout the reductive deligation process, which we term *in operando* EIS. This approach enables real-time monitoring of ligand removal by extracting key parameters such as the charge-transfer resistance. By systematically varying applied potential and pH, we gain kinetic and mechanistic insight into Au_25_ deligation and provide experimental evidence that protons play an important role in this transformation. Ultimately, this study establishes *in operando* EIS as a powerful electrochemical characterization tool for monitoring *in situ* catalyst evolution and deepens our understanding of Au_25_ deligation behavior.

## Abstract 2

*In operando* electrochemical impedance spectroscopy provides *in situ* monitoring of the dynamic evolution of gold nanoclusters, offering mechanistic insight and kinetic quantification of their catalytic activation process: reductive deligation.

## Graphical abstract

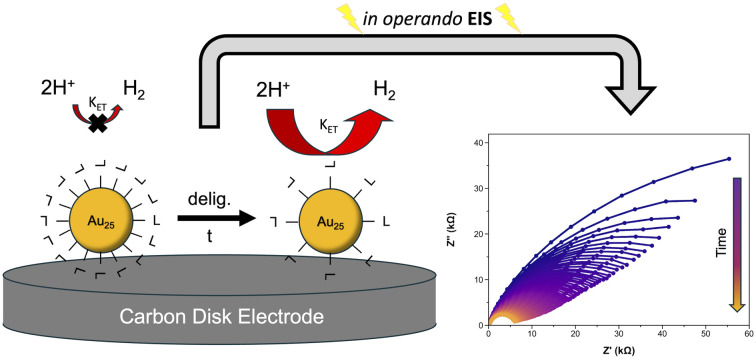

## Introduction

Understanding catalyst evolution is fundamentally important in nanomaterial catalysis, given that catalytic reactions occur on dynamic active sites that evolve throughout the reaction process.^1–3^ However, monitoring catalyst evolution remains challenging, as commonly used *ex situ* characterization techniques only provide snapshots of the catalyst composition at the start and end point of the reaction, failing to capture the dynamic changes that occur during the process.^3–5^ More specifically, many nanomaterial catalysts undergo an activation step—a specific form of catalyst evolution—where the initially synthesized material transforms into its catalytically active form.^6,7^ A well-known example is Ni(OH)_2_ in oxygen evolution reactions (OER), where the true active catalyst is not the as-prepared Ni(OH)_2_ but the NiOOH species formed *in situ* during the reaction.^8–10^

Recently, atomically precise metal nanoclusters have emerged as promising catalysts due to their ultrasmall size and highly strained, undercoordinated surface sites.^11,12^ Among them, gold nanoclusters (AuNCs) have been investigated for catalytic applications such as carbon dioxide reduction (CO_2_RR) and the hydrogen evolution reaction (HER).^11,13–16^ To synthesize these structurally well-defined clusters, organic ligands—typically thiolate ligands—are needed to stabilize the nanocluster and precisely control the morphology and nuclearity.^17,18^ However, these ligands also passivate the surface by blocking access to active metal sites. Consequently, fully-ligated nanoclusters are generally considered electrocatalytically inactive. Ligand removal is therefore a critical step in catalyst activation that has been investigated through both computational and experimental approaches.^19–21^ Nevertheless, the detailed mechanisms underlying nanocluster deligation remain poorly understood. Mpourmpakis and co-workers, through computational studies, suggested that cleaving the S–R bond is thermodynamically more favorable than cleaving the Au–S bond in Au_25_(SR)_18_ nanoclusters (SR = thiolate ligands).^22^ In contrast, a recent report by Lee and co-workers indicated that the deligation of AuNCs occurs *via* the removal of the thiolate group, implying Au–S bond cleavage.^21^ Sun *et al.* used advanced first-principle calculations to show that protons in the aqueous solutions play a key role in the deligation process.^23^ Furthermore, Tang and co-workers demonstrated computationally that the pH of the aqueous environment directly affects ligand detachment dynamics.^24^ Despite these important contributions, the deligation of gold nanoclusters has not been thoroughly investigated experimentally, particularly due to a lack of time-resolved, *in situ* characterization. As a result, a kinetic understanding of the deligation process during catalysis remains limited.

There have been significant efforts to develop novel methods for probing electrocatalyst evolution, which include liquid cell transmission electron microscopy (LC-TEM), differential electrochemical mass spectroscopy (DEMS), and *in situ* infrared and Raman spectroscopy methods.^25–32^ These methods are often costly, technically demanding, and not widely accessible. Rather than relying on costly and technically demanding spectroscopic techniques in conjunction with electrochemistry to achieve time-resolved insights into material evolution, electrochemical methods themselves can serve as powerful tools for *in situ* characterization during electrocatalysis.^33^ Previously, electrochemical impedance spectroscopy (EIS) has been utilized for kinetic monitoring in biosensor systems, characterization of the solid electrolyte interphase (SEI) in batteries, and investigation of corrosion processes.^34–41^ In this work, we successfully apply EIS to track dynamic catalyst evolution during electrocatalysis *in situ*, a method we term *in operando* EIS. Using this approach, we investigated the well-studied [Au_25_(PET)_18_]^−^ (PET = 2-phenylethanethiolate) nanocluster to gain a kinetic understanding of the ligand removal process.^42^ The hydrogen evolution reaction (HER) served as a probe to track the extent of gold active site exposure throughout the deligation process. By performing experiments at various deligation potentials, we determined that there is a potential window where increasingly reducing applied potentials accelerated the deligation rate, consistent with the Butler–Volmer equation.^43,44^ Additionally, we varied the pH of the solution to study the influence of proton concentration on the deligation process. We demonstrate experimentally that the deligation process is strongly influenced by proton concentration, suggesting a proton-coupled mechanism, as previously predicted theoretically. Lastly, we show that increased HER activity at the gold active sites can compete with the deligation process.

## Results and discussion

**Fig. 1:**
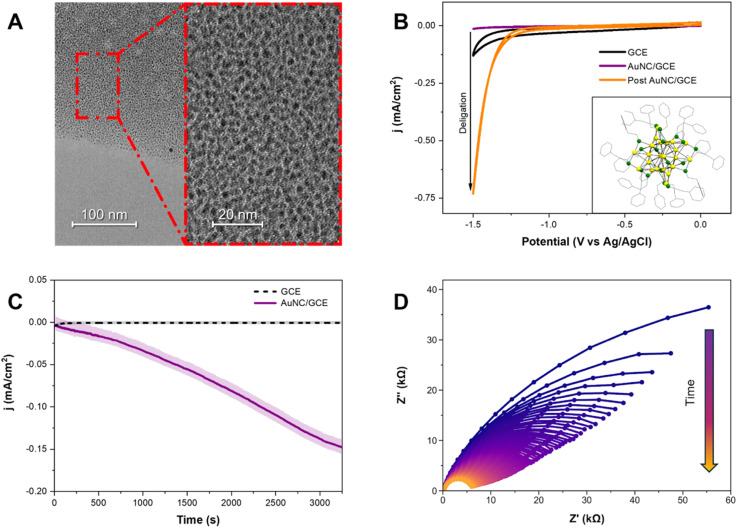
Au_25_ nanoclusters undergo reductive deligation, which can be tracked *via in operando* electrochemical impedance spectroscopy. (A) Transmission electron microscope image of spuncoat Au_25_ on a TEM grid, each nanocluster can be clearly differentiated, and a monolayer film of Au_25_ can be seen. (B) Experimental cyclic voltammograms collected in 100 mM KCl at 50 mV s^−1^ with a blank glassy carbon electrode (GCE), and Au_25_/GCE pre and post deligation at −1.3 V *vs.* Ag/AgCl, represented with black, purple, and orange, respectively. The inset shows an Oak Ridge Thermal–Ellipsoid Plot (ORTEP) of Au_25_ with Au (gold) and S (green) atoms (CCDC code: 691773).^56^ The carbon atoms are represented in gray, and all hydrogen atoms, the toluene solvate, and the cation are omitted for clarity. (C) *I*–*t* trace during the deligation process at −1.3 V *vs.* Ag/AgCl for AuNC/GCE (purple, solid) and bare GCE (black, dashed), indicating increased HER activity throughout the process on AuNC/GCE, whereas no activation on the GCE control. The solid curves are the moving average of the data over 33 points, while the lighter curves are the raw data. (D) *In operando* EIS spectra, with an applied DC voltage of −1.3 V *vs.* Ag/AgCl, and an AC waveform amplitude of 10 mV with a frequency range of 1 MHz to 5 Hz, measured sequentially during the reductive deligation process. The first cycle is represented in purple and sequential scans are shown with a purple-orange gradient; the first 100 cycles are shown. The system's resistance goes down with time, indicating the removal of ligands.

The [Au_25_(PET)_18_]^−^ nanoclusters (hereafter referred to as Au_25_) were synthesized following a previously reported procedure.^45^ The Au_25_ was characterized with proton nuclear magnetic resonance spectroscopy (^1^H NMR) and electrospray ionization mass spectrometry (ESI-MS) to confirm its purity and stability (Fig. S1 and S2). The crystal structure of the Au_25_ nanoclusters is displayed in the Fig. 1B inset.^46^ This cluster features an Au_13_ centered icosahedral core and an outer [Au_12_(PET)_18_] shell consisting of both gold atoms and 2-phenylethanethiolate ligands. For electrochemical experiments, the Au_25_ solvated in toluene (2 mg mL^−1^) was spin-coated at 1000 rpm onto a glassy carbon working electrode (GCE) to form Au_25_/GCE. X-ray photoelectron spectroscopy was performed onto the Au_25_/GCE film, confirming that the spin-coated Au_25_/GCE film maintains its purity (Fig. S5).^21^ Transmission electron microscopy image shows the homogeneity of the film, and the uniformity of the ultra-small Au_25_ nanoclusters (*r* = 0.2613 ± 0.090 nm, *N* = 485) (Fig. 1A).

The electrochemical ability of Au_25_ towards catalyzing hydrogen evolution reaction (HER) was first characterized by cyclic voltammetry. As seen in Fig. 1B, the HER activity of the thiolate-protected Au_25_ nanoclusters (purple trace) is significantly reduced compared to the bare glassy carbon electrode (black trace), indicating that the surface becomes electrochemically inert upon modification. This difference is attributed to the formation of an insulating layer of Au_25_ clusters, which passivates the electrode. A concurrent decrease in CV's capacitance relative to the unmodified electrode further confirms successful deposition of the non-conductive material. The observed reduction in conductivity arises from the insulating nature of the Au_25_ ligands, which, when uniformly drop-cast onto the electrode, form a surface that is both electrically insulating and catalytically inactive.

To activate these nanoclusters for electrocatalytic HER, we perform a reductive deligation step to remove the protecting thiolate ligands – a process that is the focus of this work.^20^ We performed the deligation step by holding the electrode at −1.3 V *vs.* Ag/AgCl while rotating at 1600 rpm for 1 hour, using a rotating disk electrode (RDE) (Fig. S3). To ensure the stability of the reference electrode during this process, we verified that the Ag/AgCl reference did not drift using the redox behavior of hexammine ruthenium(iii) as a control (Fig. S4). The potential of −1.3 V *vs.* Ag/AgCl was selected as the representative deligation potential, as it falls within the range previously reported for the electrochemical reductive deligation of the Au_25_ nanoclusters. This potential also represents the threshold before hydrogen evolution becomes significant on the surface during deligation, causing visible bubble formation.^20,21^ Using RDE and high rotational speed is important for two reasons. First, it ensures a constant and high mass transport of analyte (hydronium in this case) towards the surface. Secondly, the fast rotation process mitigates any hydrogen bubbles that form at the surface of the electrode throughout the deligation process. After the deligation process, we took a cyclic voltammogram of the Au_25_/GCE surface (Fig. 1B, orange). The cathodic current at −1.5 V *vs.* Ag/AgCl, indicative of HER activity, has increased by more than 50-fold, reflecting significant catalyst evolution and activation by partial removal of the thiolate ligands from the nanocluster surface.

Although the capacitance of the deligated Au_25_/GCE increased relative to the pristine (pre-deligation) surface, it remained substantially lower than that of the bare GCE, implying that the Au_25_ film remains largely intact with no delamination. This is supported by X-ray photoelectron spectroscopy (XPS), which confirms the continued presence of both gold and sulfur on the surface after deligation (Fig. S5 and S6). Next, we compare the post Au_25_/GCE cyclic voltammogram to the voltammogram of a bare gold electrode (Fig. S7). Notably, the onset potential for HER shifted positively to approximately −1.2 V *vs.* Ag/AgCl, about 200 mV more anodic than the onset on the bare GCE. This is consistent with the activity towards HER of a bare gold electrode, confirming that HER activity arises from exposed gold active sites.^47^ To further confirm that the HER activity originates from exposed gold and not from the underlying GCE, we subjected a bare GCE to reductive polarization at −1.3 V *vs.* Ag/AgCl for 1 hour and compared its cyclic voltammograms before and after the treatment (Fig. S8). The voltammograms showed no significant changes in the electrochemical behavior of the GCE surface following polarization. These results confirm that the observed HER activity stems from electrochemical deligation of the Au_25_ nanoclusters, which exposes catalytically active gold sites by stripping away the ligand shell.

To understand the kinetics of the deligation process, we first examined the chronoamperometric trace during the process, as shown in Fig. 1C. The raw *i*–*t* trace data is shown in gray, and the moving average of the raw data is plotted by the black line. For comparison, the *i*–*t* trace for a bare glassy carbon electrode subjected to the same reductive treatment is shown as a dashed line. This confirms that the glassy carbon electrode itself undergoes no activation under these conditions. The *i*–*t* trace confirms that the deligation process is a continuous process, as the reductive current, which represents HER activity, increases seemingly sigmoidally. These observations imply that the deligation process follows a sigmoidal growth behavior, where initially of deligation on the Au_25_ surface is slow, but accelerates as ligands are removed before saturating as more and more ligands are removed. However, the current alone cannot be directly correlated with any physical properties of the Au_25_ film. To tackle this problem, we use an electrochemical technique known as electrochemical impedance spectroscopy (EIS), which applies a small sinusoidal potential over a wide frequency range while measuring the current response. Because processes at the electrode surface operate on different time scales, EIS can separate faster events which appear at high frequencies from slower ones which appear at low frequencies. Analyzing the impedance response allows us to resolve the resistance and capacitance of each electrochemical process, providing direct insight into the surface's physical and electrical properties. Furthermore, we can perform sequential electrochemical impedance spectroscopy during the reductive deligation process, which we termed *in operando* EIS. This approach enables continuous monitoring of the surface's physical characteristics while simultaneously applying the reductive potential required for ligand removal.

EIS data are commonly shown as Nyquist plots, with the real impedance on the *x*-axis and the negative imaginary component on the *y*-axis. Electrochemical processes often appear as semicircular arcs that can be modeled by RC (resistor–capacitor) circuits, where the semicircle diameter reflects charge-transfer resistance. Deviations from an ideal semicircle indicate non-ideal capacitive behavior, consistent with constant phase element characteristics rather than a perfect capacitor.

The Nyquist plots from the first 100 cycles are shown in Fig. 1D, where the initial cycle is represented in purple, and sequential scans are represented on a purple-orange gradient. Over successive cycles, we observe a clear decrease in the system's total impedance, demonstrated by the reduction of the RC circuit's semicircle diameter, indicating increased electrochemically active surface area and enhanced HER activity due to the progressive removal of thiolate ligands. For comparison, the Nyquist plot of a bare gold electrode (Fig. S9) displays minimal resistance, consistent with its high active surface area and inherent HER activity at these potentials. EIS spectra of bare glassy carbon electrode were obtained to determine the effects of the underlying glassy carbon electrode support (Fig. S10). The impedance of the bare glassy carbon electrode evolves over the first 6 cycles before reaching a stable state. To avoid conflating the intrinsic electrochemical behavior of the GCE under reductive potentials with that of our system, we exclude these initial cycles from all analyses.

**Fig. 2:**
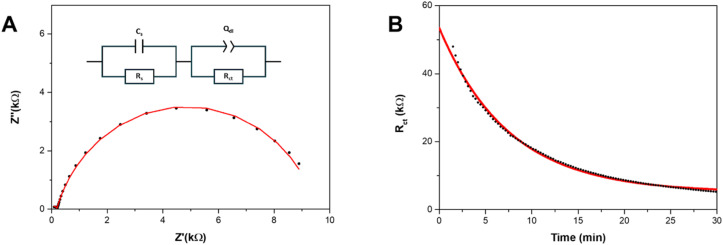
Fitting EIS spectra to the equivalent circuit enables the quantification of the charge transfer resistance over time, giving insight into the kinetic rates of Au_25_ deligation. (A) One representative EIS spectra (cycle 70) (black points), is fitted to the inset equivalent circuit (red line). (B) Equivalent circuit element *R*_ct_ (charge-transfer resistance to HER) *vs.* time (black points) is fitted to an exponential decay function (red line) to obtain a rate constant *k*_delig_ = 0.00222 s^−1^, *R*^2^ = 0.9965.

To further analyze the electrochemical processes occurring at the surface, we deconvoluted their characteristic timescales and extracted key parameters using equivalent circuit modeling. A scientifically grounded equivalent circuit was used to fit the EIS spectra at each time point, enabling quantification of the physical and electrochemical properties of the Au_25_/GCE surface. The chosen four-element model (Fig. 2A, inset) includes the solution resistance (*R*_s_), stray capacitance (*C*_s_), charge-transfer resistance (*R*_ct_) and a constant phase element (*Q*_dl_) representing the non-ideal double-layer capacitance. Although bulk solution is typically modeled as a purely resistive element, at sufficiently high frequencies, a small semicircle representing both the resistance and stray capacitance from the system can be observed.^48,49^ This stray capacitance is generally attributed to the dielectric properties of the medium or to capacitance between the reference electrode and the cell. Notably, this feature remains small and consistent across our experiments (<10 nF) and has minimal impact on the fitted values of *R*_ct_ and *Q*_dl_. Furthermore, the solution resistance remained relatively constant throughout the experiment, indicating minimal changes in local electrolyte composition or concentration (Fig. S11). The charge-transfer resistance (*R*_ct_), which reflects faradaic charge transfer within the system, can be attributed primarily to HER. The faradaic contribution from deligation is negligible compared to HER and does not significantly influence *R*_ct_. The double-layer capacitance was modeled as a constant phase element (CPE) to account for contributions from both the Au_25_ and the underlying glassy carbon electrode (Fig. S12). Because the nanocluster surface is partially insulating and dynamically evolving during the deligation process, the resulting double layer is imperfect and non-ideal. This non-ideality is captured by the CPE phase parameter, *α*, which was approximately 0.7 for the pristine surface and increased to ∼0.85 by the end of deligation (Fig. S13). While we cannot directly attribute changes in *α* to specific physical properties such as surface roughness or electronic inhomogeneity, it is well established that any surface transitioning from an insulating organic layer to a conductive metallic layer will exhibit variations in the CPE's *α*-value. A purely resistive surface corresponds to *α* = 0, whereas an ideal conductive surface yields *α* = 1.^50^ The pristine surface is expected to be both electronically inhomogeneous and resistive, due to contributions from the underlying glassy carbon electrode and the fully ligand-protected Au_25_. This results in a lower initial *α*-value. As ligands are progressively removed, the surface becomes more conductive, causing the *α*-value to approach unity. However, due to incomplete deligation and residual surface roughness, *α* never reaches 1. The selected equivalent circuit successfully fits the impedance spectra across all measured cycles during deligation. A representative spectrum and its corresponding fit are shown in Fig. 2A.

Based on this equivalent circuit, as the hydronium ions are catalyzed by the gold active sites *via* the HER reaction, we can measure the dynamic charge-transfer resistance (*R*_ct_) of the Au_25_/GCE surface throughout the deligation process. This is illustrated in Fig. 2B, where the *R*_ct_ is plotted against time. The charge transfer resistance decreases continuously throughout the deligation process, following an exponential decay trend that contrasts with the sigmoidal shape of the corresponding chronoamperogram. To explain this difference in shape and gain a deeper understanding of the specific physical characteristics reflected in the charge transfer resistance (*R*_ct_), we consider the following relationship:^51^1
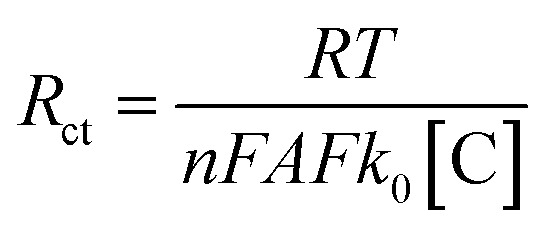
where *R* is the gas constant, *T* is temperature, n is number of electrons (1), *F* is Faraday's constant, *A* is electrochemical surface area, *k*_0_ is the heterogeneous charge transfer rate constant, and [C] is the concentration of redox species. The *k*_0_ in this case, is representative of the heterogeneous electrochemical rate constant for HER on the gold active sites formed upon removal of thiolate ligands. The contribution of the glassy carbon electrode is minimal, as the EIS spectra for a bare glassy carbon electrode remain constant (Fig. S10). While some HER may occur on the glassy carbon, any observed changes in charge transfer resistance (*R*_ct_) can be confidently attributed to the gold sites.

Although the structure of active sites may vary with the number of removed ligands, the nanoclusters are atomically precise and uniform, implying that *k*_0_ remains relatively consistent. Consequently, *R*_ct_ is inversely proportional to the electrochemically active surface area (*A*), or more specifically, the number of gold active sites (*N*_Au_), enabling us to track the number of exposed active sites using the experimentally obtained *R*_ct_. As previously observed, the *i*–*t* plots exhibit sigmoidal behavior, indicating that the number of gold active sites can fit a logistic function:2
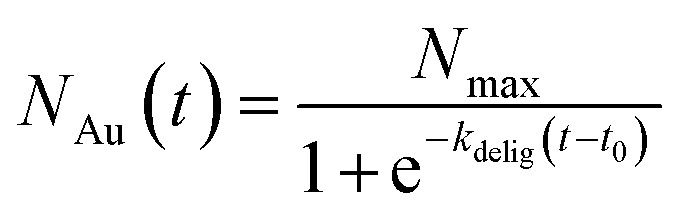
where *N*_Au_(*t*) is the number of exposed active sites at time *t*, *N*_max_ is the maximum number of exposed active sites, *k*_delig_ is the apparent deligation rate constant, and *t*_0_ is the inflection point, corresponding to the time of fastest deligation.

Given that *R*_ct_ ∝ 1/*N*_Au_, we can express *R*_ct_(*t*) in the form of an exponential decay:3
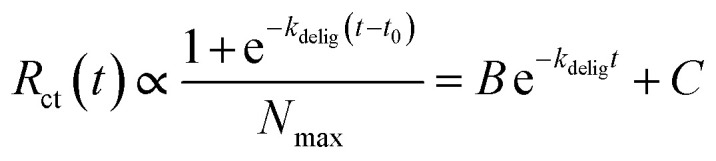


This model is supported by our experimental data, as the *R*_ct_*vs.* time plot is best described by a single exponential decay curve. Fitting this curve allows us to extract the apparent deligation rate constant, *k*_delig_, which characterizes the kinetics of the ligand removal process on Au_25_ (Fig. 1B). We are able to obtain a quantitative apparent deligation rate (*k*_delig_) by fitting the *R*_ct_ values obtained from *in operando* EIS to an exponential decay curve, which is 0.00222 ± 0.00003 s^−1^ for the deligation process at −1.3 V *vs.* Ag/AgCl plotted in Fig. 2B. This corresponds to a half-life of 312 ± 5 seconds, or just over 5 minutes. Our observed kinetics align with previously reported values for ligand-induced structural transformations in both gold and copper nanoclusters.^52,53^

**Fig. 3:**
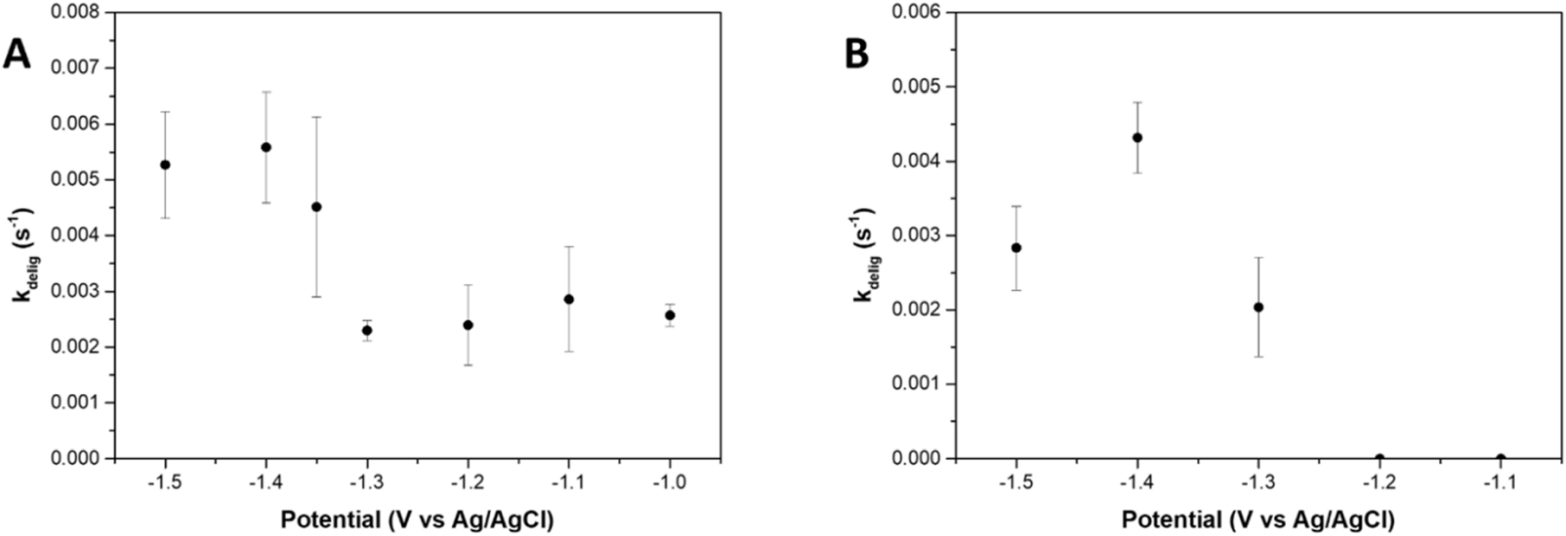
Gold nanoclusters deligation rates (*k*_delig_) determined *via in operando* EIS at different applied potentials and pH. (A) *k*_delig_ values determined by fitting *R*_ct_*vs.* time from the *in operando* EIS data obtained under neutral conditions (deaerated 100 mM KCl solution) at applied potentials of −1.0, −1.1, −1.2, −1.3, −1.35, −1.4, and −1.5 V *vs.* Ag/AgCl. (B) *k*_delig_ values obtained under unbuffered basic conditions (deaerated 10 mM KOH, 90 mM KCl solution) at applied potentials of −1.1, −1.2, −1.3, −1.4, and −1.5 V *vs.* Ag/AgCl.

To investigate the potential dependence of the deligation kinetics, we determined the deligation rate at various applied potentials *via in operando* EIS. Under neutral conditions, Au_25_ clusters were subjected to reductive potentials ranging from −1.5 V to −1.0 V *vs.* Ag/AgCl, and the deligation rate constants (*k*_delig_) were measured over three replicates at each potential (Fig. 3A and S14). Variability across these trials likely stems from subtle differences in the Au_25_ film formed on the GCE surface. Potentials outside this range either resulted in no observable deligation (below −1.0 V *vs.* Ag/AgCl) or were significantly affected by bubble formation at the electrode surface (above −1.5 V *vs.* Ag/AgCl). At potentials below −1.0 V *vs.* Ag/AgCl, no change in *R*_ct_ was observed over time, and cyclic voltammograms (CVs) of the film showed no significant increase in HER activity after 60 minutes, indicating that no deligation occurred.

At moderately reductive potentials—from −1.0 V to −1.3 V *vs.* Ag/AgCl—the deligation rate remained relatively constant, with *k*_delig_ values falling between 0.002 s^−1^ to 0.003 s^−1^. We hypothesize that, within this potential window, only the initial deligation steps are thermodynamically accessible, resulting in a plateau in the deligation rate (*k*_delig_). We attempted to use X-ray photoelectron spectroscopy (XPS) to quantify the number of ligands removed at each potential; however, the sulfur 2s region lacked sufficient resolution to reliably determine ligand loss (Fig. S6). Consequently, we were unable to further assess this low-potential region. From −1.3 V to −1.4 V, the deligation rate increased with applied potential, suggesting a potential-dependent kinetic regime, reminiscent of the empirical Butler–Volmer relationship that relates applied potential to reaction kinetics. Beyond −1.4 V *vs.* Ag/AgCl, the rate plateaued again, indicating that at more negative potentials, the deligation process is no longer limited by electrochemical driving force. Given the long experimental timescales (typically 10–60 minutes) and the high rotational speed (1600 rpm), mass transport limitations—such as proton or hydroxide diffusion—can be ruled out. Instead, we propose that at sufficiently negative potentials, the kinetic bottleneck arises either from the structural reorganization of the cluster, or from competition with HER activity at these active sites. As thiolate ligands are progressively removed, the stability of the Au_25_ core decreases, necessitating metal–ligand framework rearrangements to accommodate and stabilize the increasingly activated cluster. Additionally, at these highly negative potentials, increased HER activity may directly compete with deligation.

To investigate the role of protons as intermediates in the Au_25_ deligation process, we conducted additional deligation experiments under basic conditions. An unbuffered solution of 10 mM KOH/90 mM KCl was used, maintaining the same overall ionic concentration as in previous experiments but increasing the pH to 12 (Fig. 3B and S15). At applied potentials of −1.1 and −1.2 V *vs.* Ag/AgCl, the *R*_ct_ remained constant over time, with the *R*_ct_*vs.* time plots showing no exponential decay. This indicates minimal to no deligation occurred at these potentials.

Compared to neutral conditions, where deligation initiates at −1.0 V *vs.* Ag/AgCl, the need for more negative potentials (−1.3 V *vs.* Ag/AgCl) in basic conditions suggests that protons play a key role in the deligation of Au_25_. Specifically, the rate determining step likely involves a proton-coupled electron transfer (PCET) mechanism. Similar to neutral conditions, the deligation rate under basic conditions increased from −1.3 V to −1.4 V *vs.* Ag/AgCl, marking the potential-dependent regime. Notably, the highest deligation rate is at −1.4 V *vs.* Ag/AgCl, while the rate decreased by over two standard deviations at −1.5 V *vs.* Ag/AgCl.

We hypothesize that the observed decrease in deligation rate at highly reductive potentials is due to increased hydrogen evolution reaction (HER) activity at the Au active sites. This enhanced HER can either directly compete with the deligation process for electrons or result in the formation of H_2_ microbubbles on the electrode surface, physically blocking active sites and hindering further catalysis.

To probe this hypothesis, we reduced the rotation speed of the rotating disk electrode (RDE) from 1600 rpm to 800 rpm, effectively lowering the proton flux to the electrode surface. This reduction in mass transport limits the availability of protons, thereby modulating HER activity. Under these conditions, *k*_delig_ decreased at −1.4 V but increased at −1.5 V *vs.* Ag/AgCl (Fig. S16). This contrasting behavior suggests that at moderately negative potentials, lower proton availability slows deligation due to reduced PCET activity, whereas at more negative potentials, the suppression of excessive HER (*via* lowered proton flux) alleviates its inhibitory effect on the deligation.

**Scheme 1:**
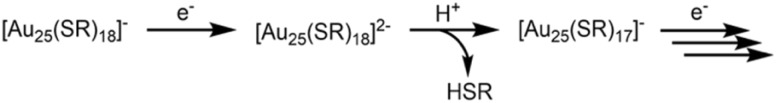
Proposed mechanism for the deligation of Au_25_ nanoclusters.

These results imply that excessive HER activity at highly negative potentials suppresses deligation process. Moreover, the potential-dependent regime of Au_25_ deligation shifts with mass transport conditions, which highlights the interplay between proton availability, HER, and deligation kinetics. Based on these observations, we propose the following mechanism for the deligation of Au_25_ nanoclusters (Scheme 1): an electron is first injected into the Au_25_ nanocluster from the electrode, forming a 2^−^ charged species. The resulting excess negative charge facilitates protonation of a –SR group. This protonation weakens the Au–S bond, leading to its cleavage and the formation of a deligated Au_25_ species. This process can then repeat, resulting in the stepwise loss of additional thiolate ligands.

## Conclusion

In this study, we establish *in operando* electrochemical impedance spectroscopy as a powerful and accessible technique for probing the dynamic structural evolution of electrocatalysts under active electrochemical conditions. Specifically, we apply this technique to monitor, in real time, the electrochemical deligation of thiolate-protected gold nanoclusters [Au_25_(PET)_18_]^−^, a process known to activate these clusters for electrocatalysis. By sequentially acquiring EIS spectra during the reductive deligation process, we extract charge transfer resistance (*R*_ct_) values that quantitatively reflect the progressive exposure of catalytically active gold sites.

Our results reveal distinct potential-dependent regimes in the deligation kinetics, with the rate of ligand removal increasing within a defined potential window, consistent with Butler–Volmer behavior. Notably, we find that the onset and rate of deligation are strongly influenced by proton availability, with more negative potentials required to initiate deligation under basic conditions, providing experimental evidence for a proton coupled deligation mechanism previously suggested by computational studies. At highly reductive potentials, the deligation rate plateaus or even decreases, which we attribute to competing hydrogen evolution reaction HER activity and structural rearrangement limitations within the nanocluster framework. From these observations, we propose a mechanism for the deligation of Au_25_ nanoclusters.

Beyond mechanistic insights into Au_25_ deligation, this work demonstrates the broader utility of *in operando* EIS as a kinetic and mechanistic probe for dynamic catalytic transformations. In this system, the kinetics are relatively slow, and thus sequential frequency scanning provides sufficient temporal resolution. Employing Fast Fourier Transform electrochemical impedance spectroscopy (FFT-EIS) can further enhance both temporal resolution and data acquisition efficiency for faster processes.^33^ Compared to traditional spectroscopic techniques, EIS offers a cost-effective, time-resolved, and surface-sensitive approach that can be applied broadly to study electrocatalyst activation and degradation processes. This methodology may serve as a valuable complement to conventional characterization tools, opening up new possibilities for understanding the evolution of catalysts under working conditions.

## Author contributions

E. Z. Liu conceptualized the project, performed the experiments, analyzed all data, and drafted the manuscript. S. D. Parker assisted with experiments, contributed to data analysis, and helped edit the manuscript. D. P. Tietje-McKinney carried out the nanocluster synthesis and contributed to manuscript editing. M. Orozco performed TEM characterization, assisted with experiments, and contributed to reviewing and editing the manuscript. T. W. Hayton and L. Sepunaru supervised the project, interpreted data, and critically revised the manuscript. All authors contributed to discussion of the data and manuscript revision.

## Conflicts of interest

There are no conflicts to declare.

## Supplementary Material

SC-OLF-D5SC05597K-s001

## Data Availability

The data supporting this article have been included as part of the supplementary information (SI).^54,55^ Additional raw data are available from the corresponding author upon reasonable request. Supplementary information is available. See DOI: https://doi.org/10.1039/d5sc05597k.
