# Supplementary material for: Rate and mechanism of thiolate deligation in Au25 nanoclusters via in operando electrochemical impedance spectroscopy
Source: Chem Sci. 2025 Nov 12;17(1):265–73. doi: 10.1039/d5sc05597k (PMC12606460; doi:10.1039/d5sc05597k)
Supplement: SC-017-D5SC05597K-s001 [file SC-017-D5SC05597K-s001.pdf]

Supplementary Information (SI) for Chemical Science.
This journal is © The Royal Society of Chemistry 2025

Supporting Information

Rate and Mechanism of Thiolate Deligation in Au_{25} Nanoclusters via In Operando Electrochemical Impedance Spectroscopy

Eric Z. Liu[a], Samuel D. Parker[a], Dylan P. Tietje-Mckinney[a], Miguel Orozco[a], Trevor W. Hayton[a], Lior Sepunaru*[a]

[a] Department of Chemistry and Biochemistry, University of California at Santa Barbara Santa Barbara, CA 93106, United States. E-Mail: sepunaru@ucsb.edu

Supporting Information

Table of Contents:

**Content**	**Page**
Experimental Procedures	S2 – 4
Characterization of Synthesized $[Au_{25}(PET)_{18}]^-$ – Figure S1	S5
Characterization of Degraded $[Au_{25}(PET)_{18}]^-$ – Figure S2	S6
Electrochemical Cell Setup – Figure S3	S7
Reference Electrode Stability – Figure S4	S7
XPS of Pristine Au25 – Figure S5	S8
XPS of Deligated Au25 – Figure S6	S8
CV of Gold Electrode – Figure S7	S9
Pre/Post CV of Glassy Carbon Electrode – Figure S8	S9
EIS of Gold Electrode – Figure S9	S10
EIS of Glassy Carbon Electrode – Figure S10	S10
R_s vs time – Figure S11	S11
C_{dl} vs time – Figure S12	S11
α vs time – Figure S13	S12
Pre/Post CV, Nyquist Plots, R vs t : Neutral – Figure S14	S13
Pre/Post CV, Nyquist Plots, Rvs t : Base – Figure S15	S14
k_{delig} at Slow Rotational Speeds – Figure S16	S15
References	S16

Experimental Section

Materials and Instrumentation

The following chemicals were purchased and used as received: potassium chloride (Sigma-Aldrich, 99.0%), potassium hydroxide (Sigma-Aldrich, 99.99%), isopropyl alcohol, phenylethane thiol (Sigma-Aldrich, 98%), gold (III) chloride trihydrate (Sigma-Aldrich, 99.9%), tetraoctylammonium bromide (Sigma-Aldrich, 98%), sodium borohydride (Sigma-Aldrich, 98%), Dichloromethane (Sigma-Aldrich, 99.8%). Toluene and hexanes were dried by passage over activated molecular sieves using a Vacuum Atmospheres DRI-SOLV solvent purification system and stored over 3 Å molecular sieves for 24 h prior to use. Tetrahydrofuran (THF) was distilled twice, first from calcium hydride and then from sodium benzophenone ketyl and stored over 3 Å molecular sieves for 24 h prior to use. CD2Cl2 was dried over activated 3 Å molecular sieves for 24 h prior to use.

Synthesis of Au 25 (PET) 18

A 250 mL round-bottom flask was charged with gold (III) chloride trihydrate (192 mg, 0.488 mmol), tetrahydrofuran (50 mL), and a stir bar. Then, tetraoctylammonium bromide (296.7 mg, 0.543 mmol) was added as a solid and let stir for 15 minutes. During this time, the solution changed colors from yellow to orange. Then, 2-phenylethanethiol (0.34 mL, 2.54 mmol) was added via syringe and let stir for 4 hours. During this time, the color turned a lighter yellow before becoming entirely colorless. At this time, the reaction flask was removed from the glovebox, and to it, a pre-chilled sodium borohydride (177 mg, 4.67 mmol) solution (5 mL DI water) was added all at once. The solution immediately turned dark brown upon addition. This was left to stir for 2 days. The stirring was then stopped, and the reaction mixture was allowed to settle. Two clear layers were observed, a red/brown THF layer and a milky white aqueous layer. The THF layer was pipetted off and filtered through a celite-supported frit, and the volatiles were removed *in-vacuo,* providing a dark red/brown oil. The product was then transferred to a conical vial with ~12 mL of methanol and washed via centrifugation. The supernatant was decanted, and this process was repeated 6 times. The product was then extracted into dichloromethane (~1 mL) via centrifugation and filtered through a celite-supported column (0.5 x 5 cm) supported by glass wool. The volatiles were removed *in-vacuo*, and the dried solid was brought back into the glove box. The product was layered with toluene (~2 mL) and hexanes (~15 mL). Storage in the freezer for 72 hours yielded dark brown solids which were isolated by decanting the supernatant, washing with hexanes (3 x 1 mL), and drying *in-vacuo* (38 mg, 26%).

Thin Film Spin-Coat Procedure

Au25 was dissolved in dry, deoxygenated toluene (2mg/mL) and stored under inert atmosphere. A 10 μL aliquot of this solution was drop-cast onto the surface of a glassy carbon rotating disk electrode (RDE) while spinning at 1000 rpm. Spin-coating was performed in ambient conditions, with the electrode maintained at the target rotational speed using the RDE motor to

ensure even radial dispersion of Au25 across the electrode surface. Solvent evaporation occurred during the spin process over ~5 minutes, yielding a thin, uniform Au25 film.

Electrochemical Experiments

A three-electrode configuration was used with a Ag/AgCl reference electrode (CH Instruments), a graphite rod counter electrode (6.15 mm diameter, Thermo Scientific), and either a glassy carbon or gold disk working electrode (Pine Research, 5mm diameter). Rotating disk electrode (RDE) experiments were performed using a Pine MSRX motor and RDE sensor, controlled via the EC-Lab software suite.

Electrolyte solutions were prepared using Milli-Q water (resistivity ≥ 18.2 MΩ·cm) to ensure minimal ionic contamination. Neutral and basic electrolyte solutions were prepared by dissolving the appropriate salts in ultrapure water to yield either 100 mM KCl or a mixture of 10 mM KOH and 90 mM KCl, respectively. All electrochemical measurements were conducted in a sealed glass electrochemical cell containing a total volume of 55 mL of electrolyte solution. Prior to each experiment, the solution was sparged with argon at a flow rate of 50 sccm for 30 minutes to remove dissolved oxygen, and a continuous argon flow was maintained throughout the measurements to maintain an inert atmosphere.

All the following experiments were referenced against Ag/AgCl and used a glassy carbon disk electrode and graphite counter electrode and a floating output. Before experimentation, the glassy carbon electrode was polished in 1, 0.3, and 0.05 µm diamond slurry for 2 min at each size, sonicated in a 1:1 mixture of ultrapure water and isopropyl alcohol, rinsed with ultrapure water, and dried using a lint-free Kimwipe. All impedance measurements were done with a RDE rotating at a rate of 1600rpm. Measurements spanned the frequency range from 5 Hz to 1 MHz, with an AC potential magnitude of 10 mV. Cyclic voltammograms were collected before and after each EIS experiment from 0 V to –1.5 V vs Ag/AgCl at a scan rate of 50 mV/s under stationary conditions.

The conversion from Ag/AgCl reference to reversible hydrogen electrode (RHE) reference is + 0.611 V at pH 7, and + 0.906 at pH 12. -1.3 V vs Ag/AgCl would be - 0.689 V vs RHE at pH 7 and - 0.393 V vs RHE at pH 12.

Spectroscopic characterization of gold nanoclusters:

^{1}H NMR spectra were recorded on a Varian UNITY INOVA 500 MHz spectrometer. ^{1}H NMR spectra were referenced to external $SiMe_4$ using the residual proton solvent peaks as internal standards.

Mass spectra were collected at the Materials Research Laboratory Shared Experimental Facilities at the University of California, Santa Barbara, using an electrospray ionization (ESI) source in negative ion mode with a Waters Xevo G2-XS TOF Time-of-Flight mass spectrometer. Model mass spectra were generated in MassLynx V4.1 software with the isotope clusters displayed with a minimum abundance of 0.1%

X-ray Photoelectron Spectroscopy (XPS) measurements were collected on the ThermoFisher ESCALAB Xi+ XPS system with a monochromatic Al Kα X-ray source (1486.6 eV). The spot size diameter is 650 μm and the Au25/GCE was used directly as the sample. All binding energies were calibrated against the C 1s peak at 285 eV, and all data was smoothed.

Transmission electron microscopy (TEM) imaging was obtained on a Thermo Scientific Talos F200X (scanning) transmission electron microscope operated at 200 kV with a single-tilt sample holder. For characterization, TEM grids (item numbers 01816 and 01895) were purchased from Ted Pella. Samples were prepared by spin-coating Au25 (2mg/mL) onto a TEM grid. AI-enhanced nanoparticle analysis was used to obtain the size distribution for the Au25.[1,2]

A

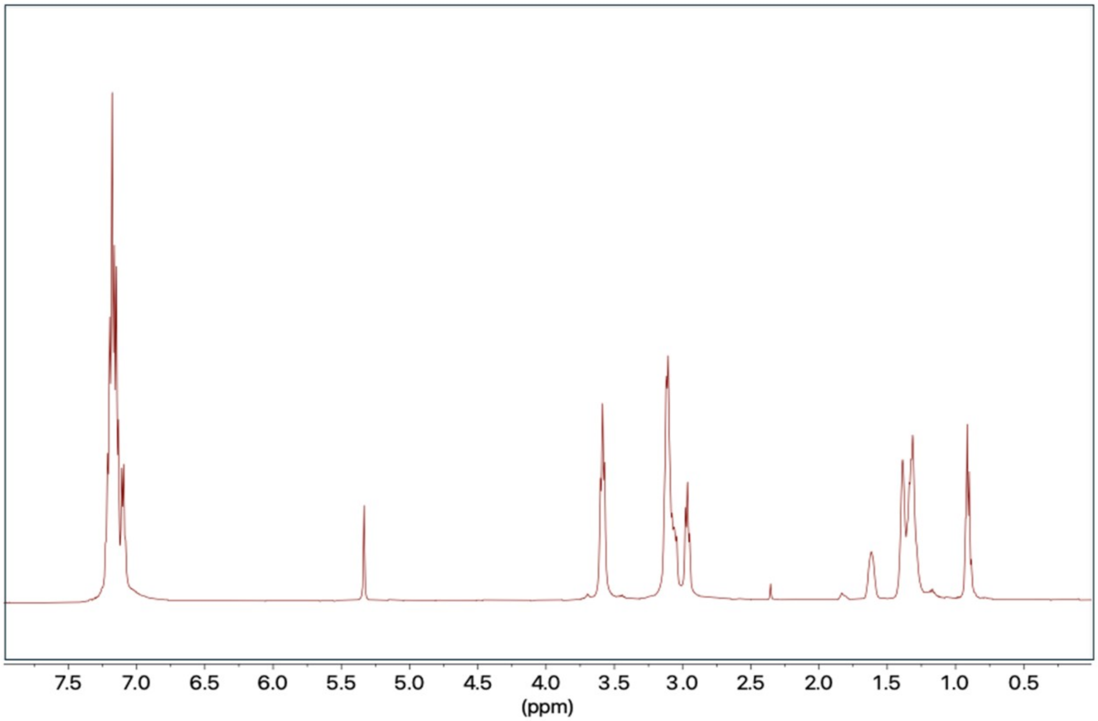

B

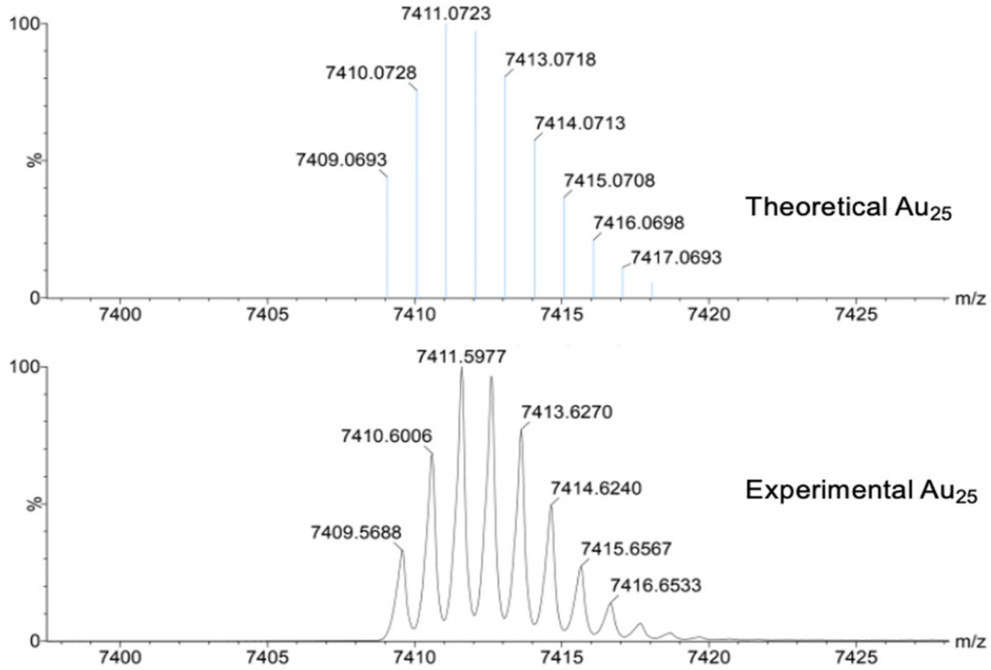

Figure S1. Characterization techniques confirm the identity of synthesized $[Au_{25}(PET)_{18}]^{-}$. A) 1H NMR spectrum of Au25 in CD2Cl2. B) Partial ESI-MS spectrum (negative ion mode, 2.50 kV) spectrum of Au25. The top spectrum is simulated, and the bottom spectrum is observed

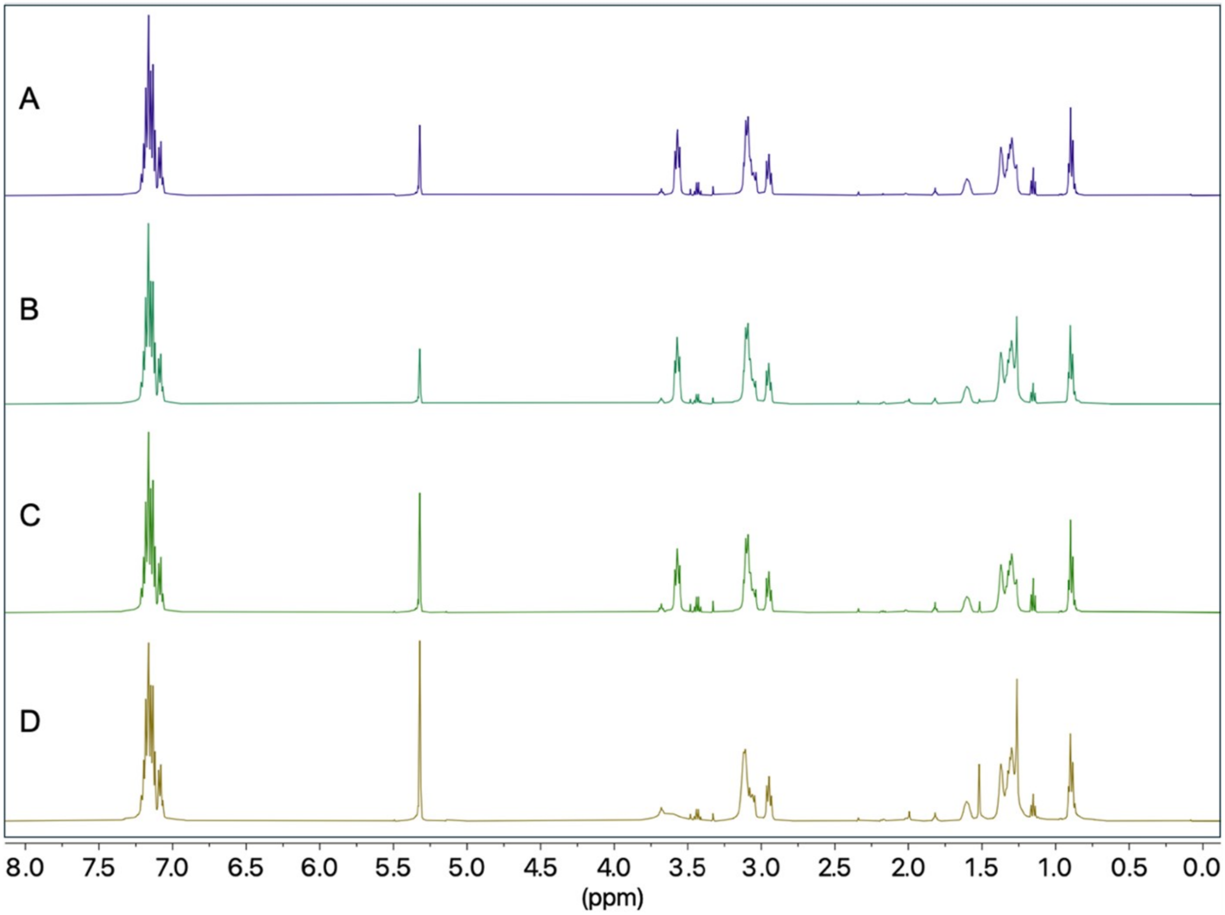

Figure S2. NMR studies indicate that a solution of Au25 (3.2 mg/0.4 mL) in CD_2Cl_2 remains stable for at least 16 hours when not exposed to air and for at least 1 hour after exposure to air. A) Initial, no exposure to air B) 16 hours, no exposure to air C) 1 hour, exposure to air D) 4 hours, exposure to air

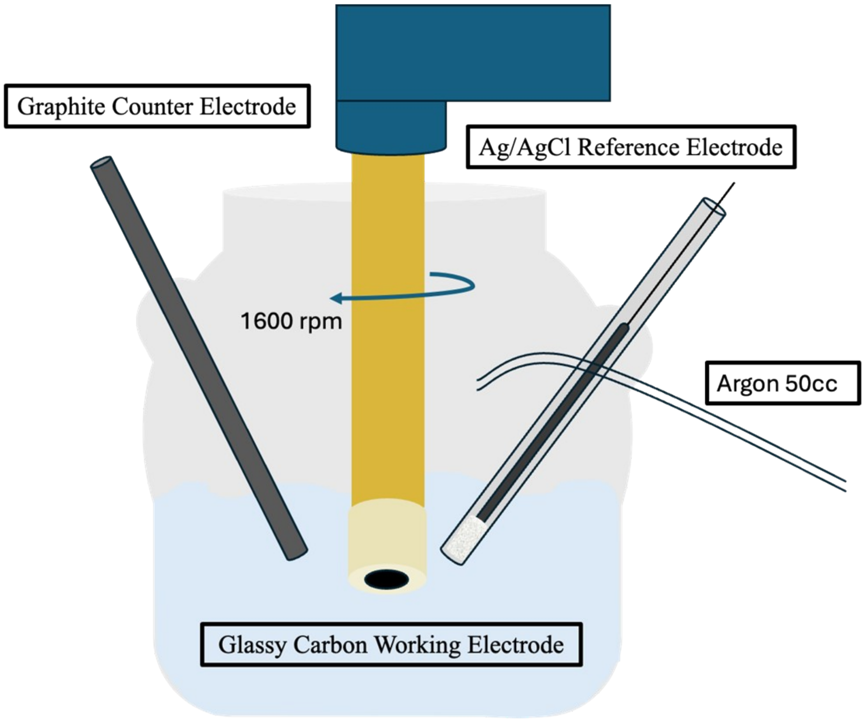

Figure S3. Diagram of electrochemical setup for *in-operando* EIS with rotating disk electrode.

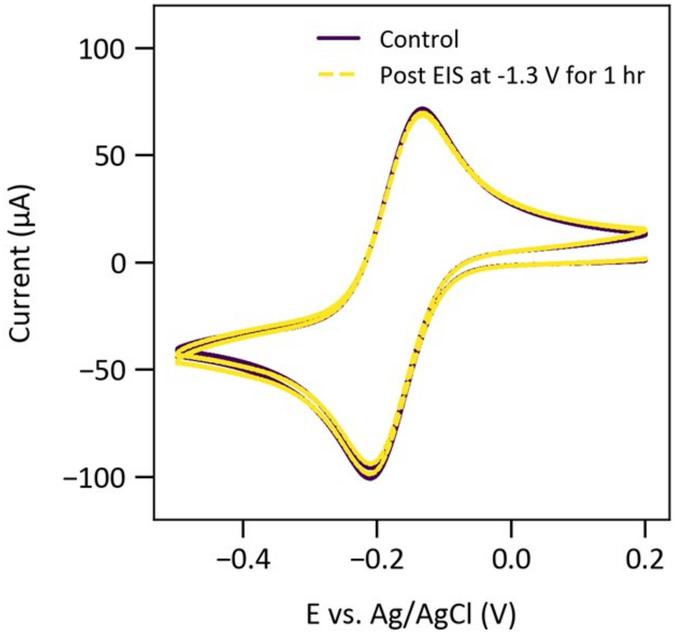

Figure S4. Ag/AgCl reference electrode stability confirmed with redox behavior of hexammine ruthenium (III). Cyclic voltammogram of 3mM hexammine ruthenium (III) in 100mM KCl at scan rate 50 mV s^{-1} taken on GCE before and after 1 hour of reductive polarization at -1.3 V vs Ag/AgCl.

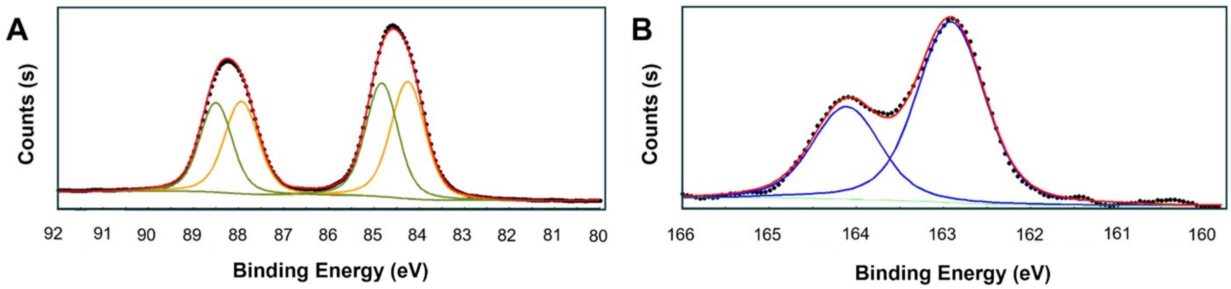

Figure S5. XPS spectra of pristine Au25/GCE. A) Au 4f and B) S 2p . Experimental data shown in black dots, with component fitting of the 4f for Au(I) (green) and Au(0) (orange) and 2p for S (blue) with the total fit in red.

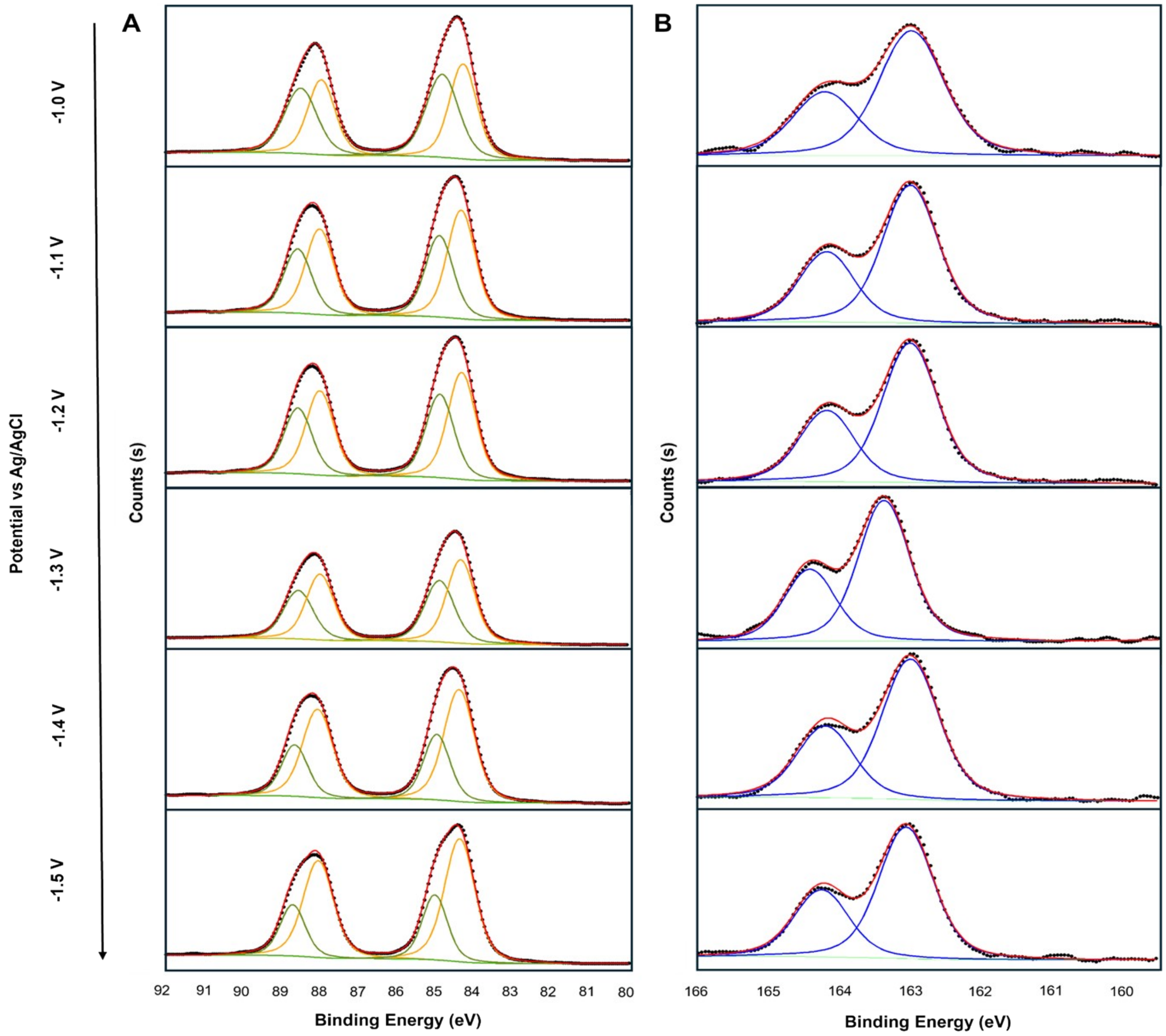

Figure S6. XPS spectra of deligated Au25/GCE. A) Au 4f and B) S 2p . Experimental data shown in black dots, with component fitting of the 4f for Au(I) (green) and Au(0) (orange) and 2p for S (blue) with the total fit in red. All deligation processes were performed at the listed potential for 30 minutes in 100 mM KCl.

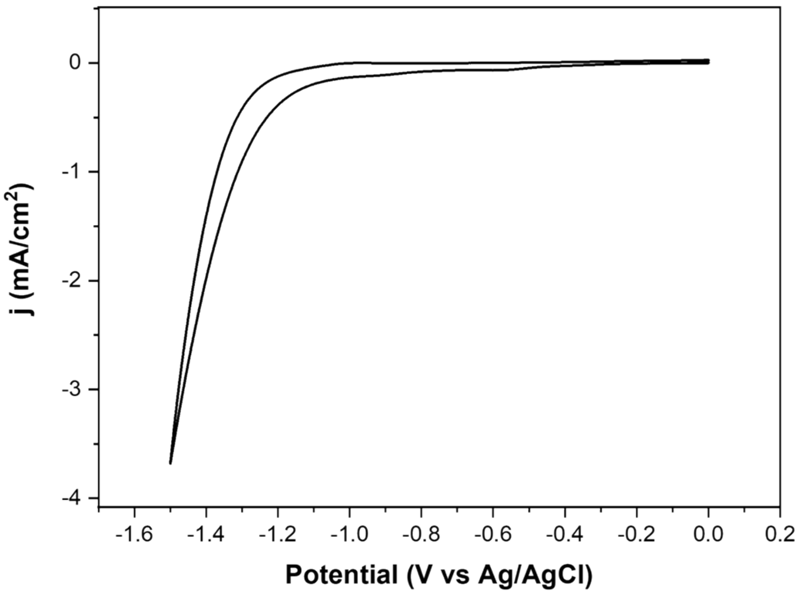

Figure S7. Cyclic voltammogram of bare gold electrode. Cyclic voltammogram collected in 100mM KCl at scan rate 50mV s^{-1}

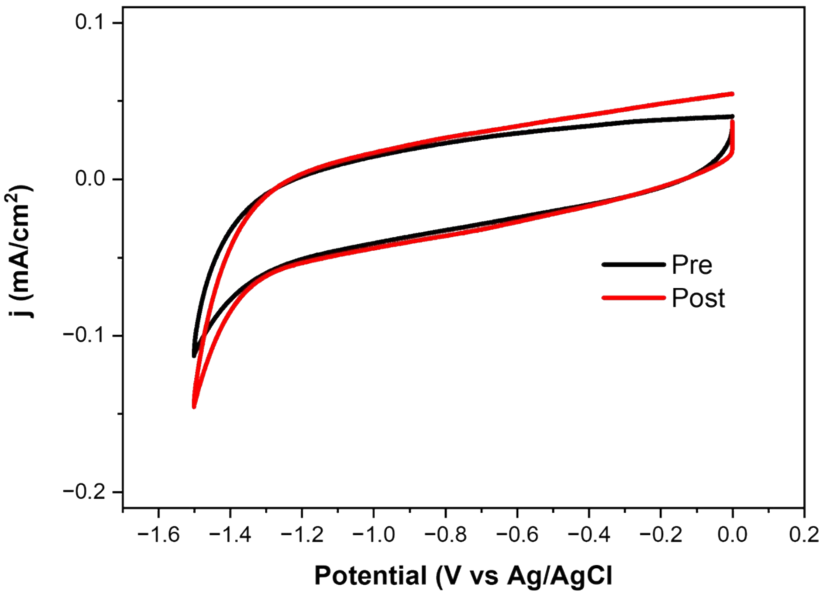

Figure S8. Cyclic voltammograms of a bare glassy carbon electrode before and after reductive polarization. Cyclic voltammograms collected of the bare glassy carbon electrode before and after 1 hr of reductive polarization at -1.3 V vs Ag/AgCl, showing no significant changes of the bare glassy carbon electrode from reductive polarization. Cyclic voltammograms collected in 100mM KCl at scan rate 50 mV s^{-1}.

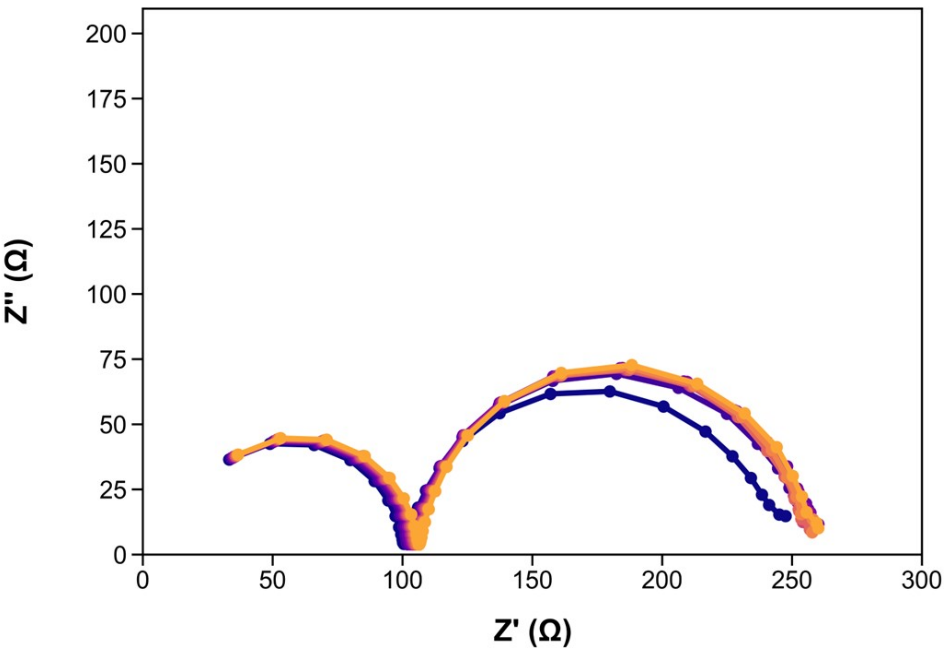

Figure S9. Nyquist plot of bare gold electrode. EIS data collected with DC voltage of -1.5 V vs Ag/AgCl, AC waveform amplitude of 10 mV, and a frequency range of 1MHz to 5 Hz. A total of 10 cycles were collected, with the cycles represented in a purple to orange gradient.

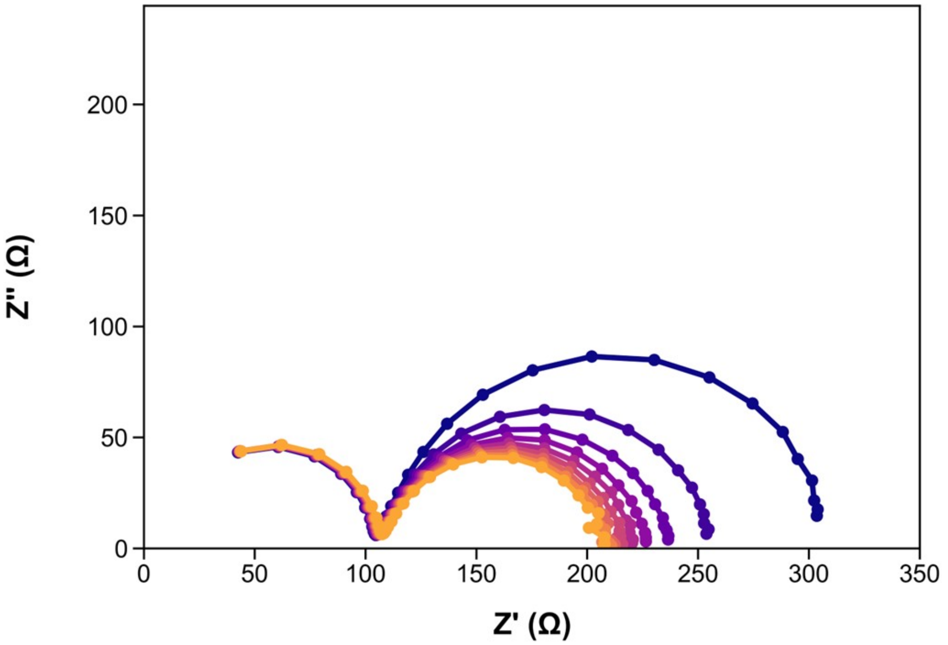

Figure S10. Nyquist plot of bare glassy carbon electrode. EIS data collected with DC voltage of -1.5 V vs Ag/AgCl, AC waveform amplitude of 10 mV, and a frequency range of 1MHz to 5 Hz. A total of 10 cycles were collected, with the cycles represented in a purple to orange gradient. The EIS curves are stable after 6 cycles.

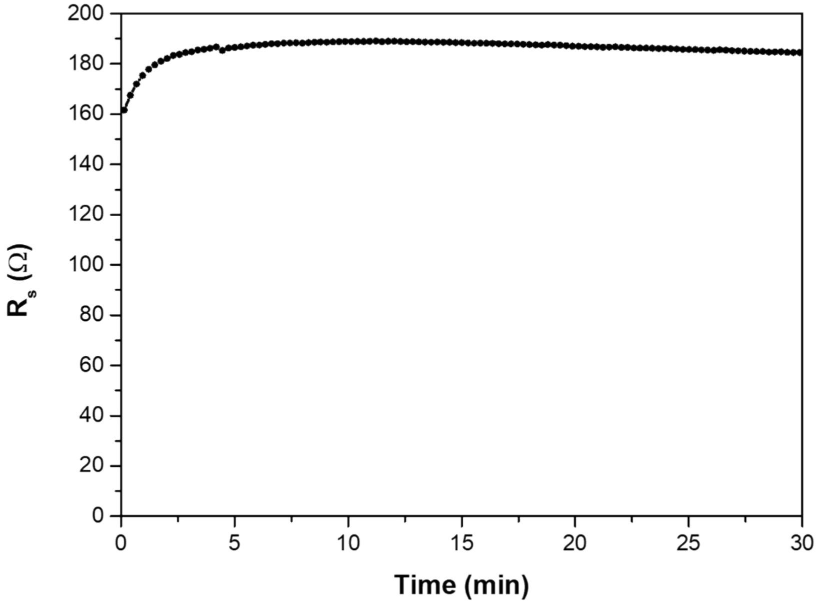

Figure S11. R_s vs time at -1.3V vs Ag/AgCl under neutral conditions.

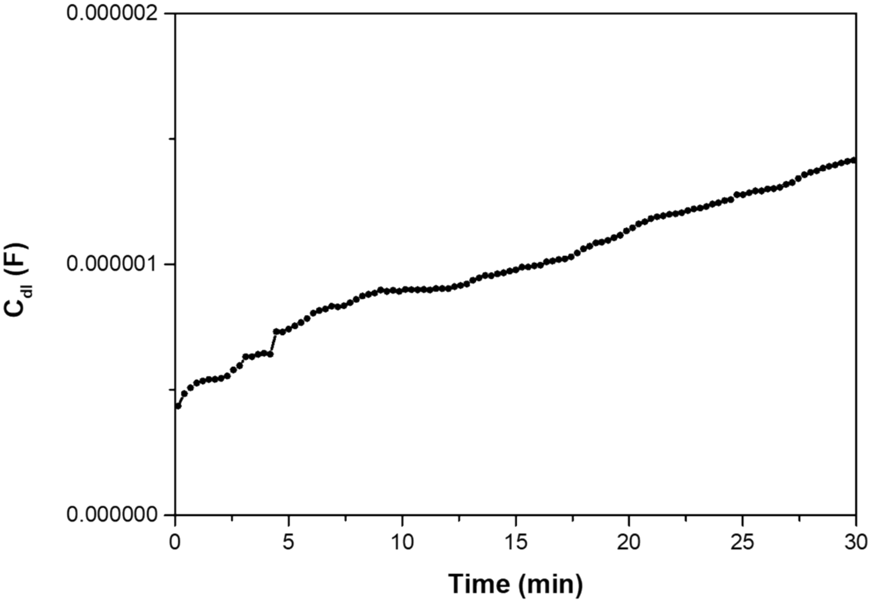

Figure S12. C_{dl} vs time at -1.3 V vs Ag/AgCl under neutral conditions.

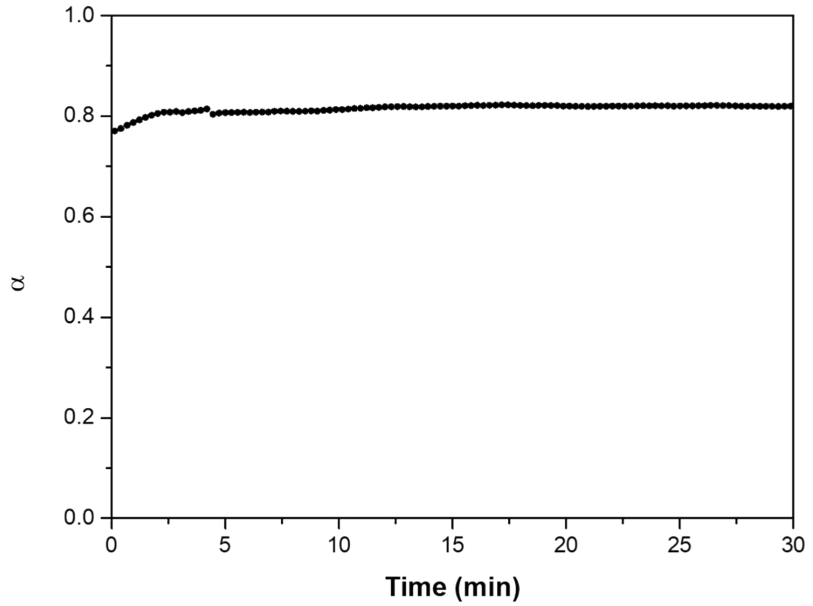

Figure S13. α vs time at -1.3 V vs Ag/AgCl under neutral conditions.

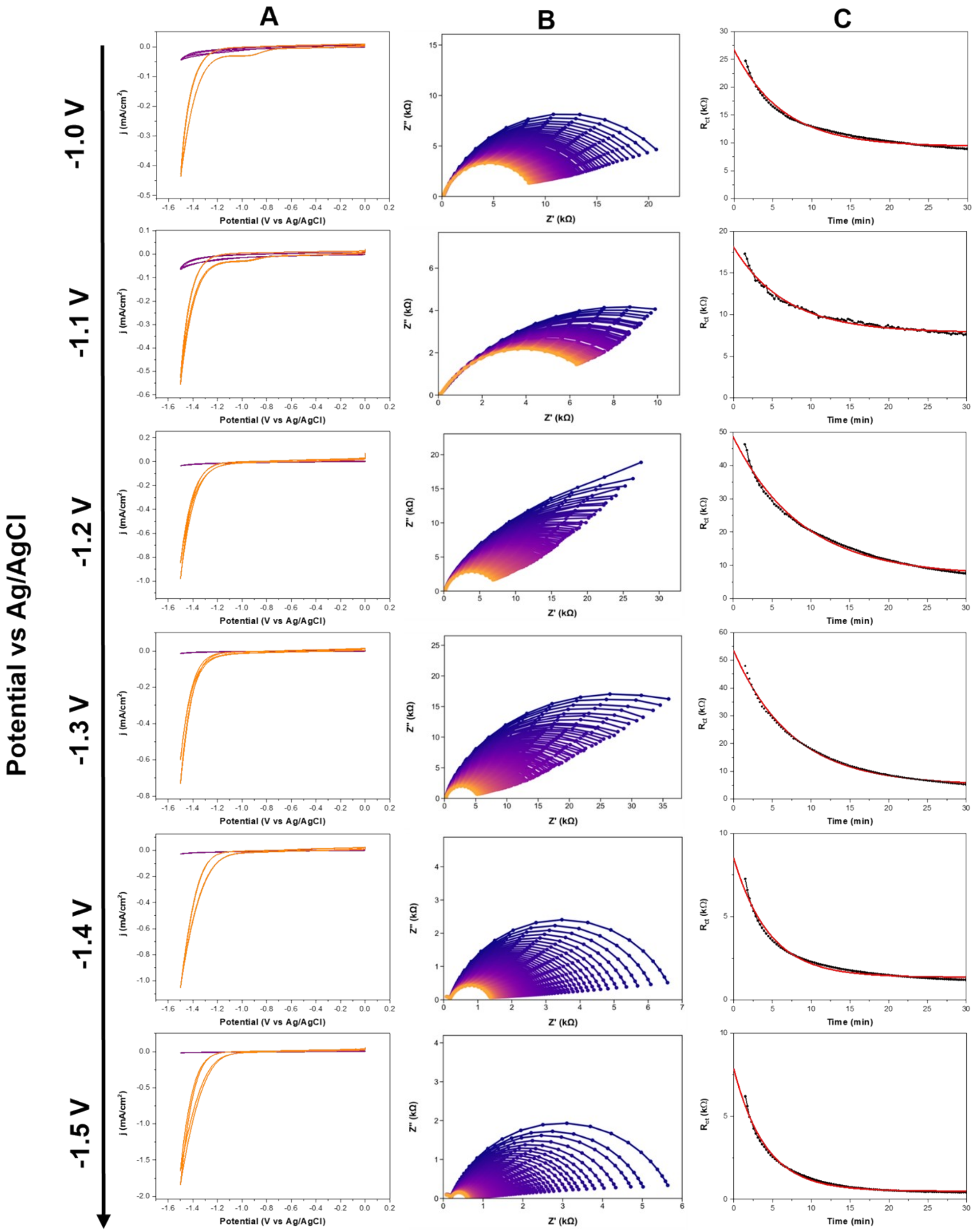

Figure S14. A) Cyclic voltammograms collected in 100mM KCl at 50 mV s^{-1} with Au25/GCE pre and post deligation in purple and orange, respectively. B) in operando EIS spectra, with an AC waveform amplitude of 10 mV and a frequency range of 1 MHz to 5 Hz. Cycles 7 to 112 (purple to orange). C) Resistance of charge transfer versus time shown with fit overlayed in red.

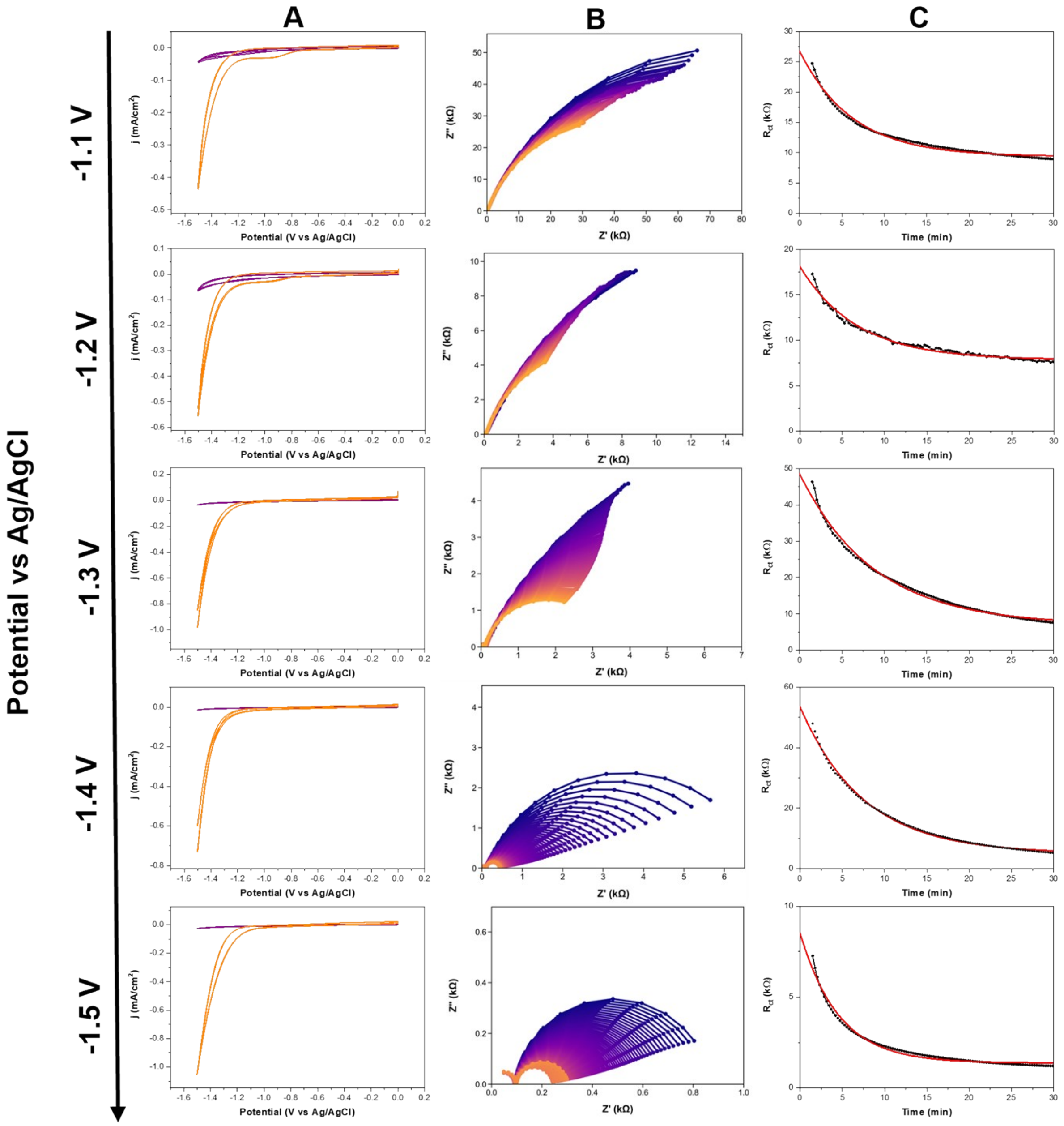

Figure S15. A) Cyclic voltammograms collected in 10mM KCl, 90mM KOH at 50 mV s^{-1} with Au25/GCE pre and post deligation represented in purple and orange, respectively. B) in operando EIS spectra, with an AC waveform amplitude of 10 mV with a frequency range of 1 MHz to 5 Hz. Cycles 7 to 112 are shown (purple to orange). C) Resistance of charge transfer versus time shown with fit overlayed in red.

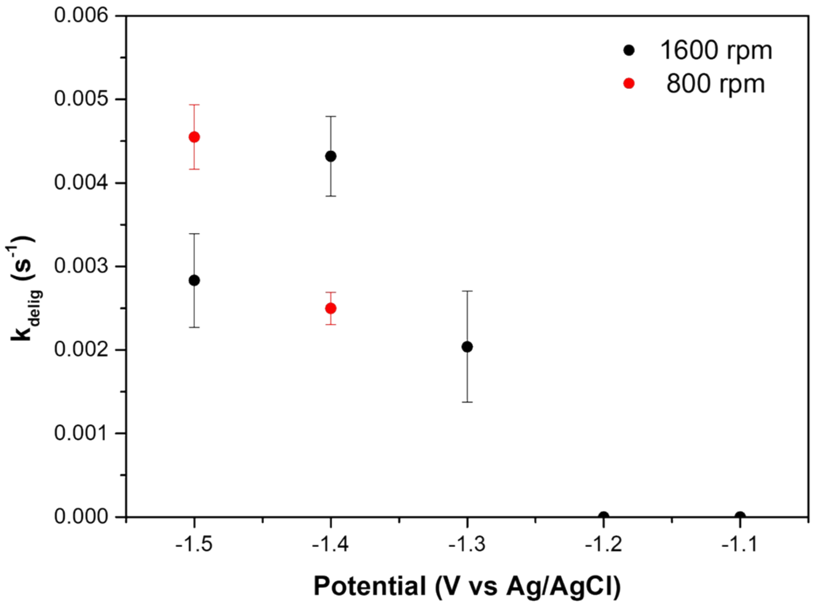

Figure S16. Gold nanocluster deligation rates (k_{delig}) at different rotational speeds. k_{delig} rates obtained at 800 rpm (red) at -1.4 and -1.5 V vs Ag/AgCl compared to the k_{delig} rates obtained at 1600 rpm (black). All rates were obtained under unbuffered basic conditions (deaerated 10 mM KOH, 90 mM KCl solution).

References:

[1] A. Genc, J. Marlowe, J. Finzel, P. Christopher, *Microsc. Microanal.* **2024**, *30*, ozae044.196.

[2] A. Genc, J. Marlowe, A. Jalil, D. Belzberg, L. Kovarik, P. Christopher, *Ultramicroscopy* **2025**, *271*, 114116.
